# Diabetes mellitus service preparedness and availability: a systematic review and meta-analysis

**DOI:** 10.3389/fendo.2024.1427175

**Published:** 2024-07-19

**Authors:** Melsew Setegn Alie, Desalegn Girma, Amauel Adugna, Yilkal Negesse

**Affiliations:** ^1^ Department of Public Health, School of Public Health, College of Medicine and Health Science, Mizan-Tepi University, Mizan-Aman, Ethiopia; ^2^ Department of Midwifery, College of Medicine and Health Science, Mizan-Tepi University, Mizan-Aman, Ethiopia; ^3^ Department of Public Health, College of Medicine and Health Science, Debre Markos University, Debre Markos, Ethiopia

**Keywords:** availability, preparedness, diabetes mellitus, DM, low and middle countries

## Abstract

**Background:**

In areas with limited resources, the lack of preparedness and limited availability of diabetes mellitus services in healthcare facilities contribute to high rates of illness and death related to diabetes mellitus. As a result, this study focused on analyzing the combined prevalence of preparedness and availability of diabetic services in countries with limited resources.

**Methods:**

A comprehensive search was conducted across various databases, such as PubMed/MEDLINE, Web of Science, Google Scholar, and African Journal Online. The search aimed to identify primary research articles that assessed the availability and preparedness of services for individuals with type 2 diabetes mellitus specifically. The articles included in the search spanned from January 2000 to 23 February 2024. To analyze the data, a meta-analysis of proportions was performed using the random-effects model. Additionally, the researchers assessed publication bias by examining a funnel plot and conducting Egger’s test. Heterogeneity and sensitivity analyses were also conducted to evaluate the data. The findings of the study regarding the pooled prevalence of diabetes service preparedness and availability, along with their corresponding 95% confidence intervals, were presented using a forest plot.

**Results:**

A comprehensive analysis was conducted on 16 research articles that focused on service preparedness and 11 articles that examined service availability. The sample sizes for these studies were 3,422 for service preparedness and 1,062 for service availability. The findings showed that the pooled prevalence of diabetes service preparedness was 53.0% (95% CI: 47.0-60.0). Furthermore, in this systematic synthesis, the overall pooled prevalence of service availability for diabetes mellitus was 48% (95% CI: 36.0-67.0), with the highest pooled prevalence observed in Asia, with a pooled prevalence of 58% (95% CI: 38.0-89.0).

**Conclusion:**

Our study reveals a significant disparity in the preparedness and availability of services for diabetes mellitus, which falls below the minimum threshold set by the WHO. These findings should capture the attention of policymakers and potentially serve as a foundation for reevaluating the current approach to diabetes service preparedness and availability. To enhance the availability and preparedness of diabetes services, a tailored, multifaceted, and action-oriented approach to strengthening the health system is required.

**Systematic Review Registration:**

https://www.crd.york.ac.uk/prospero/, identifier CRD42024554911.

## Introduction

Diabetes mellitus (DM) is a highly prevalent non-communicable disease (NCD) that poses a significant risk of premature death and disability (1–3). Diabetes increases vulnerability to other NCDs by changing metabolic processes (4, 5). As a result, diabetes mellitus has become a pressing and costly public health concern, prompting global leaders to take action. The diabetes burden has increased globally from time to time. According to the International Diabetes Federation (IDF), approximately 463 million adults worldwide are affected by diabetes, with 79.4% residing in low- and middle-income countries (6–8).

Diabetes is a major contributor to severe health issues, such as renal failure, limb amputation, blindness, and cardiovascular disease. These complications not only result in physical suffering and disability but also have a profound effect on individuals’ socioeconomic status. Managing diabetes can also lead to financial difficulties and jeopardize economic well-being. Additionally, these complications can impede individuals’ capacity to work and be productive. However, adequate preparedness and access to resources can mitigate the impact of these problems (9–13).

Previous studies have revealed a significant prevalence of undiagnosed diabetes (14–17). This highlights the urgent need to prioritize the diagnosis and treatment of diabetes in order to address the detrimental impact it has on health and mortality rates (18). Regrettably, a substantial number of individuals in the surveyed countries still remain undiagnosed with diabetes. This can be attributed to various factors such as limited access to healthcare services, lack of availability, and underutilization of existing resources. These factors contribute to the higher prevalence of undiagnosed diabetes in these regions (19). Furthermore, the investigated countries showed a concerning trend where only a small percentage of diagnosed patients were receiving adequate care. This emphasizes the importance of improving the overall healthcare system’s preparedness to provide comprehensive diabetes management services (20).

Achieving universal access to NCD services requires the active engagement of all stakeholders (21). Increasing investment in prevention and accessing care is essential for the achievement of Universal health care (UHC) (22). Multi-sectorial programs were required to deliver successful services for NCD. In addition, the reduction of premature death by one-third is the targeted of sustainable development goal (23, 24). Existing evidence suggests that timely provisions of such interventions to patients are excellent economic investments in reducing future expensive treatments (21, 24).

Insufficient access to healthcare services, limited availability, and underutilization of healthcare resources are major factors that contribute to the high morbidity and mortality rates associated with diabetes mellitus. Moreover, there is a pressing need to enhance service preparedness and availability, as previous studies have highlighted significant variations in the prevalence of preparedness and availability of services for diabetes mellitus (13, 18, 25–38). However, it is important to note that previous research in this field has often relied on a single study or has been limited to local or regional samples. To obtain a more comprehensive understanding, it is crucial to conduct multi-country studies that can compare the ability of healthcare facilities to provide diabetes mellitus services across countries. By addressing these limitations and conducting multi-country studies, we can gain more reliable and comprehensive insights into the preparedness and availability of healthcare facilities to provide diabetes mellitus services in low-resource settings. Therefore, the objective of this study is to synthesize the pooled prevalence of service availability and readiness for diabetes mellitus in resource-limited countries.

## Methods

### Research questions

To conduct a systematic review, we aimed to determine the pooled prevalence of diabetes service availability and readiness in resource-limited countries across Africa and Asia. Our research question followed the CoCoPop format, with the condition (Co) being the health facilities or responsible individuals involved in providing diabetic services. The context (Co) focused on health facilities in resource-limited settings. The population (Pop) of interest for this systematic review and meta-analysis consisted of adults requiring screening in health facilities within these resource-limited countries. The outcome of interest was the readiness and availability of health facilities to provide diabetic services. Consequently, our research question was formulated as follows: “What is the pooled prevalence of diabetes service preparedness and availability in resource-limited settings?” This approach enabled us to identify relevant keywords and construct comprehensive search strategies for conducting a thorough literature review.

### Protocol and registration

This study has been officially registered with the International Prospective Register of Systematic Reviews. The PROSPERO registration number is CRD42024554911.

### Data sources and search strategy

This systematic review and meta-analysis was conducted in accordance with the Preferred Reporting Items for Systematic Reviews and Meta-analyses (PRISMA) reporting guideline (39). We performed an extensive literature search using various electronic bibliographic databases such as PubMed/MEDLINE, Web of Science, Google/Google Scholar, and African Journal Online. Our main objective was to obtain accurate and reliable results so we employed a combination of keywords and a relevant thesaurus that focused on the readiness and availability of health facilities to deliver diabetic services in resource-limited countries. To identify publications discussing availability and preparedness of health facilities for diabetes mellitus, we used the following search terms: (“readiness or availability or preparedness”) AND (“diabetes mellitus” OR “DM”) AND (“Services”) AND (“Africa” or “Asia”). We included observational studies in this systematic review and meta-analysis. We independently screened titles, abstracts, and full texts of articles. In cases of disagreement regarding the inclusion of a full-text article, all authors participated in discussions to reach a consensus. To ensure transparency and adherence to a standardized approach, we developed and registered a review protocol.

### Eligibility criteria

#### Inclusion criteria

This review followed a systematic process that involved one approach, namely CoCoPop (Condition, context, and population), to determine which studies to include. Papers that failed to meet these criteria were considered irrelevant and excluded from the review. The review only took into account papers published in English from January 2000 to February 2024, including observational studies. To assess the quality of each article, the Joanna Briggs Institute (JBI) critical appraisal tool was used, and all articles that passed the quality assessment were included in the review.

#### Exclusion criteria

We have excluded studies pertaining to service availability and preparedness that primarily concentrate on communications, and reviews, commentaries, letters to the editors, studies where the raw data cannot be analyzed, and protocols were excluded. In addition, studies that did not assess the overall readiness and had unclear outcomes were also excluded from this systematic review and meta-analysis.

### Study data management

After conducting a comprehensive search and gathering multiple articles, we proceeded to eliminate any duplicate files. This screening process involved two stages: initially assessing the titles and abstracts, followed by conducting a full-text screening. To ensure accuracy, two independent reviewers utilized the EndNote software to evaluate the potential relevance of each article for further review. The assessment was based on a predefined set of inclusion and exclusion criteria. In cases where there were discrepancies between the reviewers’ assessments, they were resolved through discussion and by seeking input from a third reviewer. For the purpose of auditing, electronic records were maintained for both the included and excluded studies, with clear explanations provided for any exclusions made.

### Quality assessment and risk of bias

To assess the risk of bias in the study, a quality assessment checklist for prevalence studies was employed. This checklist, developed by Hoy and colleagues, consists of nine items that are crucial in evaluating the quality of a study (40). These items include the target population, sampling frame, sampling method, response rate, data collection procedures, study case definition, study instruments, and parameters for the numerator and denominator. Each item contributes to a total score of 9. Based on the scores obtained, the studies were categorized as having a high-risk (0–3), moderate-risk (4–6), or low-risk (7–9) of bias. Each study underwent an independent evaluation, and the majority of them demonstrated a low risk of bias. To ensure the reliability of the results, studies with a high risk of bias were excluded from the final analysis.

### Data extraction

The data extraction process utilized a Microsoft Excel template. The form underwent iterative testing and revision as necessary. Two authors, YN and DG, independently performed the extraction. In cases where there were disagreements between the data extractors, the principal author, MS Alie, facilitated discussions to resolve them. The extracted descriptive variables encompassed various aspects such as first author, country, region, study design, study period, data collection method, sample size, outcomes, response rate, and related outcomes of diabetes service availability and preparedness.

### Sensitivity analyses

A thorough sensitivity analysis was conducted to assess how individual studies affected the overall estimation of prevalence. Each study was methodically removed, and the resulting impact on the estimate was carefully examined. Surprisingly, the exclusion of any single study did not have a significant effect on the pooled prevalence estimate. Furthermore, none of the studies fell outside the confidence interval’s lower and upper boundaries. These findings indicate that the collective results of the studies remained strong and consistent, reinforcing the reliability of the overall prevalence estimate.

### Data synthesis and analysis

The data collected from various articles was processed using Microsoft Excel 2013 and then exported to R software version 4.3.2 for further analysis. Our analysis focused on examining individual studies to determine the overall prevalence, service availability, and preparedness of health facilities in delivering diabetic care services. To achieve this, we performed random-effects meta-analyses in R software, allowing us to estimate the pooled prevalence along with 95% confidence intervals (C.I.s). The results were presented using the logit transform of the individual studies, accompanied by their respective 95% confidence intervals. In this systematic review and meta-analysis, we conducted subgroup analysis, bias assessment, sensitivity analysis, and heterogeneity analysis. Given the expected variation among the studies, we employed a random-effects model and utilized I^2^ statistics to evaluate the level of heterogeneity. Specifically, I^2^ values of 25%, 50%, and 75% indicated low, medium, and high heterogeneity (41), respectively. To assess publication bias, we examined the distribution of studies in a funnel plot. Deviation from a symmetrical funnel shape can indicate the presence of publication bias. Furthermore, we performed subgroup analysis based on potential sources of heterogeneity. Additionally, we conducted a leave-one-out sensitivity analysis to assess the influence of individual studies on the overall effect, which is presented in the tables and figures.

## Results

### Search and eligible research reports

**Figure 1:**
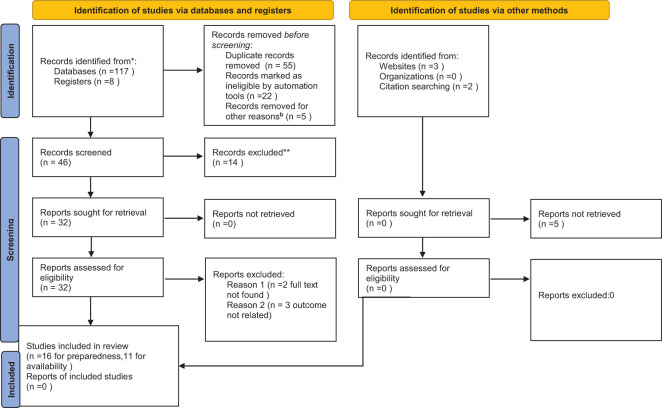
PRISMA 2020 flow diagram for diabetes mellitus service availability and preparedness in resource limited countries. ^**^protocol, communication letter, not in study area and unrelated findings, other reasons=out of study area. * indicates the articles not included in this systematic review and meta-analysis.

A total of 117 studies were browsed from PubMed/MEDLINE, Web of Science, Google/Google Scholar, African Journal Online. From a register, we found 8 articles, from websites we found three articles, and through citation searching, we found two articles. Of these studies, 84 studies were excluded due to duplication and not being in the study area. Of the remaining 46 articles, 14 articles were removed due to them being a short communication letter, protocol, or qualitative study or having unclear and unrelated outcomes. The remaining 32 articles were assessed by reviewing the full text while five articles were excluded due to outcome measurement not being related and the full text not being available to see the detailed methodology. Finally, a total of 27 studies were eligible and included in the final systematic review and meta-analysis. After careful evaluation, we found that 16 articles for diabetes service preparedness (13, 13, 28, 29, 31–34, 42–49) and 11 articles for diabetes services availability (30, 42, 45, 48, 50–56) were included in this systematic review and meta-analysis. For a visual representation of the detailed inclusion and exclusion criteria used in the study, please refer to **Figure 1**, displayed below as the PRISMA diagram.

### Characteristics of the studies

This analysis encompasses a total of 27 studies conducted in various countries. Of these, 16 studies focused on preparedness for managing diabetes, while 11 studies examined the availability of diabetes management services. Among the studies that explored diabetes service preparedness, five (31.25%) were carried out in Bangladesh, two studies were conducted in Ethiopia and Tanzania, and single studies were conducted in Zambia, Uganda, Kenya, Nepal, Cameroon, and Burkina Faso. The detailed studies on diabetes service preparedness are presented in **Table 1**. Regarding the aspect of diabetes service availability, two studies were conducted in Ethiopia, Vietnam, India and Kenya, and only one study conducted in Tanzania, Malawi, and Burkina Faso as shown in **Table 2**. The earliest survey was conducted in 2014, while the most recent one took place in 2023. In both cases cross cross-sectional studies were included. The studies that examine diabetes service preparedness are presented in **Table 1** and studies examine diabetes service availability are presented in **Table 2**.

**Table 1:** Studies that examine diabetes mellitus service preparedness.

Authors (year)	Country	Region	Study year	Sample size	Study design	Response rate	Sampling method	Number of cases	Prevalence
Getachew et al. (2017) (42)	Ethiopia	Africa	2016	547	cross sectional	100%	Stratified random sampling	290	53.0%
Jahan F et al. (2023) (43)	Bangladesh	Asia	2017	62	cross sectional	100.00%	Stratified random sampling	38	60.62%
Kabir et al. (2023) (44)	Bangladesh	Asia	May and October 2021	126	cross sectional	100.00%	Stratified random sampling	88	70.0%
Biswas T et al. (2018) (13)	Bangladesh	Asia	2014	319	cross sectional	100.00%	Stratified random sampling	159	49.80%
Cissé K, et al. (2023) (45)	Burkina Faso	Africa	2018	794	cross sectional	98.90%	Stratified random sampling	326	41.10%
Ateudjieu J et al. (2018) (46)	Cameroon	Africa	May to July 2016	100	cross sectional	100.00%	Stratified random sampling	37	37.00%
Acharya K. et al. (2019) (47)	Nepal	Asia	2017	963	cross sectional	100.00%	Simple random Sampling	655	68.01%
Ammoun et al. (2022) (48)	Kenya	Africa	June 2019 and December 2020	258	cross sectional	100.00%	Multi-stage sampling	183	71.0%
Alam W et al. (2020) (28)	Bangladesh	Asia	2018	24	cross sectional	100.00%	Simple random Sampling	4	17.20%
Biswas T, et al. (2018) (13)	Bangladesh	Asia	2018	319	cross sectional	100.00%	Stratified random sampling	185	58.10%
Akinwumi et al. (2023) (49)	Nigeria	Africa	February and April 2018.	56	cross sectional	100.00%	Multi-stage sampling	24	42.28%
Bintabara et al. (2020) (33)	Tanzania	Africa	2014–2015	1188	cross sectional	100.00%	Stratified random sampling	619	52.10%
Robert P. et al (2014) (31)	Tanzania	Africa	November 2012 and May 2013	335	cross sectional	100.00%	Simple random Sampling	187	56.0%
Isadru et al. (2021) (32)	Uganda	Africa	July 2016	148	cross sectional	100.00%	Simple random Sampling	106	71.70%
Mutale et al. (2018) (29)	Zambia	Africa	September to October 2017	46	cross sectional	100.00%	Conventional sampling	6	13.04%
Bekele et al. (2017) (34)	Ethiopia	Africa	2014	873	cross sectional	100.00%	Stratified random sampling	515	59.00%

**Table 2:** Studies that examine diabetes mellitus service availability.

Authors (year)	Country	Region	Study year	Sample size	Study design	Response rate	Sampling method	Number of cases	Prevalence
Mulugata et al.(2022) (50)	Ethiopia	Africa	February 2021–July 2021	82	cross sectional	98.80%	Multistage cluster sampling	23	28.0%
Getachew et al.(2017) (42)	Ethiopia	Africa	2016	547	cross sectional	100%	Stratified sampling	121	22.0%
KAUR et al.(2022) (51)	India	Asia	2015 and 2018	156	cross sectional	100%	Simple random sampling	50	32.0%
Cissé K, et al.(2023) (45)	Burkina Faso	Africa	2018	794	cross sectional	98.90%	Stratified sampling	429	54.0%
Ammoun et al.(2022) (48)	Kenya	Africa	June 2019 and December 2020	258	cross sectional	100%	Multistage sampling	212	82.2%
Ashigbie et al.(2020) (52)	Kenya	Africa	September 2016	59	cross sectional	100%	Multistage sampling	10	16.1%
Adinan et al.(2019) (30)	Tanzania	Africa	March to July 2017	43	cross sectional	100%	Multi-stage stratified random sampling	34	79.0%
Lutala et al.(2023) (53)	Malawi	Africa	July to early September 2021	34	cross sectional	100%	Conveniently selected	22	63.7%
Pallavi Shukla et al.(2023) (54)	India	Asia	2023	54	cross sectional	100%	Multistage Randomly sampling	45	84.0%
Duong, David B. (2015) (55)	Vietnam	Asia	January and April 2014	89	cross sectional	100%	Simple random sampling	47	53.0%
Duong et al.(2018) (56)	Vietnam	Asia	January and April 2014	89	cross sectional	100%	Simple random sampling	69	78.0%

### The pooled prevalence of service preparedness for DM

**Figure 2:**
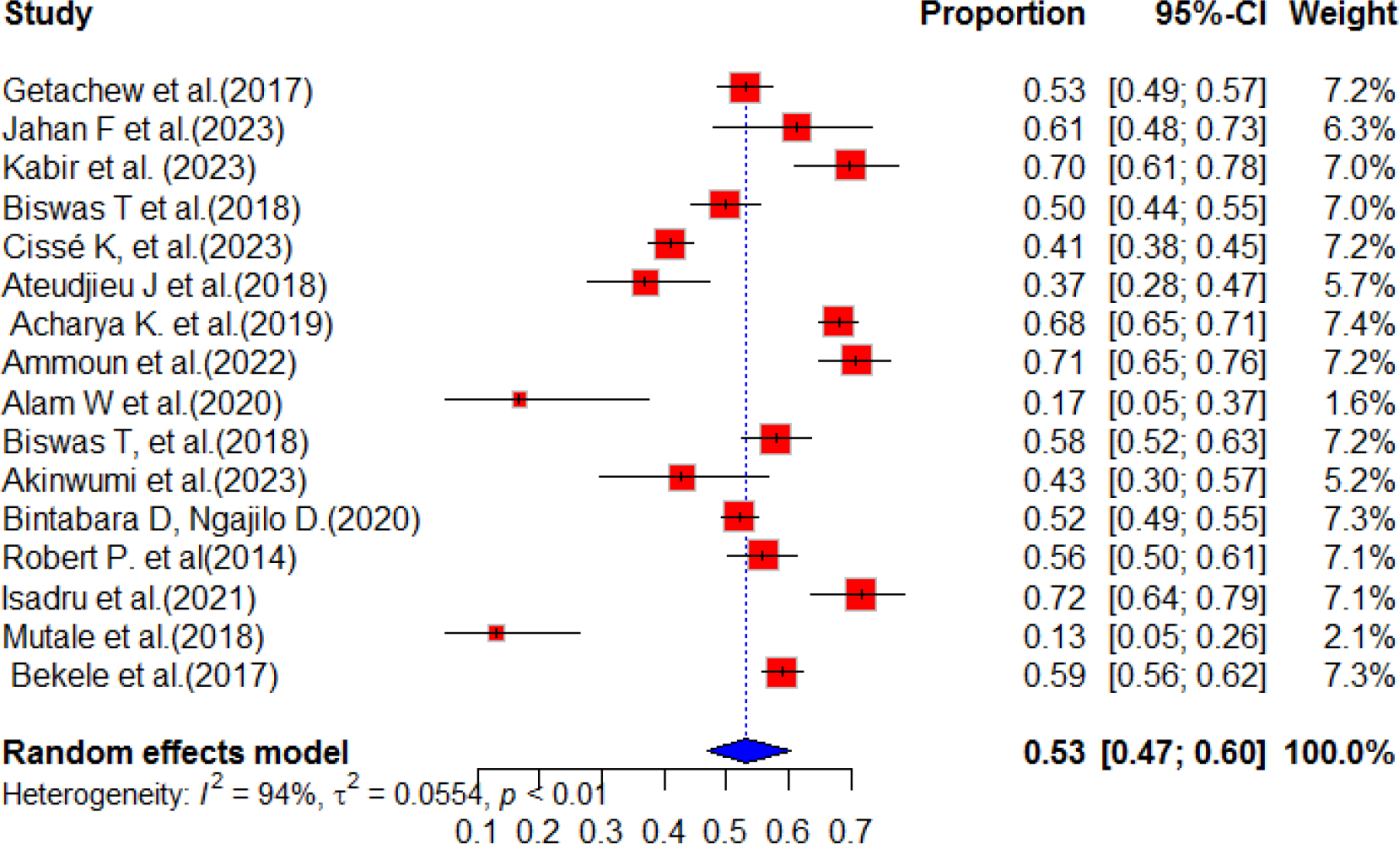
Random forest for DM service preparedness of health facilities in resource-limited countries.

This systematic review and meta-analysis was conducted to assess the availability and preparedness of diabetes services in resource-limited countries. The study analyzed a total of 26 publications from different geographical areas, including Ethiopia, Zambia, Morocco, Bangladesh, Uganda, Kenya, Nepal, Tanzania, and Nigeria. The findings revealed that the estimated pooled prevalence of diabetes service preparedness was 53.0% (95%CI; 47.0-60.0), with a p-value of less than 0.01 and a high level of heterogeneity (I^2^ = 94%) (**Figure 2**). **Figure 2** presents the pooled prevalence of diabetes mellitus service preparedness in health facilities.

### Meta-bias in prevalence of diabetes service preparedness

**Figure 3:**
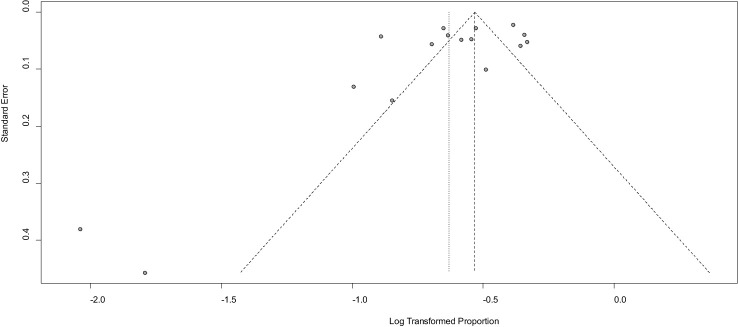
Funnel plot showing publication bias among the studies used to compute diabetes service preparedness in resource-limited countries, 2024.

The studies’s publication bias was assessed using Egger’s test and by examining the asymmetry of the funnel plot. It is important to note that funnel plots can sometimes appear highly asymmetric, even in the absence of publication bias, due to correlations between the outcome, effect size, and its standard error. In our current systematic review and meta-analysis, the results of Egger’s test indicated a p-value of 0.1119, with a bias estimate of -3.0644 (SE = 1.8063). Furthermore, the funnel plot displayed asymmetry, as illustrated in **Figure 3**. This finding indicates that there is no publication bias for the studies. Publication bias was also checked by using sample size and publication year in **Table 3** below.

**Table 3:** Meta-regression analysis of factors affecting between-study heterogeneity in the prevalence of preparedness of diabetes mellitus services in health facilities.

Heterogeneity source	Estimate	Z-value	Std. Err.	p-value	CI	I^2^
Sample size	0.0001	0.7257	0.0002	0.4680	(-0.0002,0.0005)	97.01%
Publication year	0.0131	0.5175	0.0253	0.6048	(-0.0366,0.062)	96.88%

### Sensitivity analysis

The sensitivity analysis of the study was conducted. The random effects model was used to calculate a combined estimate, which showed significant variation in the evaluation of health facilities’ preparedness for DM services. To address this variation, sensitivity analysis and subgroup analysis were performed. These analyses aimed to understand the potential impact of individual studies on the overall estimate of the prevalence of DM service preparedness. Importantly, the results from the random effects model did not identify any studies that had an excessive influence on the overall estimate. To further investigate the source of heterogeneity, a meta-regression analysis was conducted, considering factors such as sample size and year of publication. However, the results showed that neither sample size nor year of publication had a significant effect on the heterogeneity observed between studies (**Table 3**). In addition, a sensitivity analysis for sub-group analysis by the sampling method was conducted.

### Sub-group analysis of service preparedness

**Figure 4:**
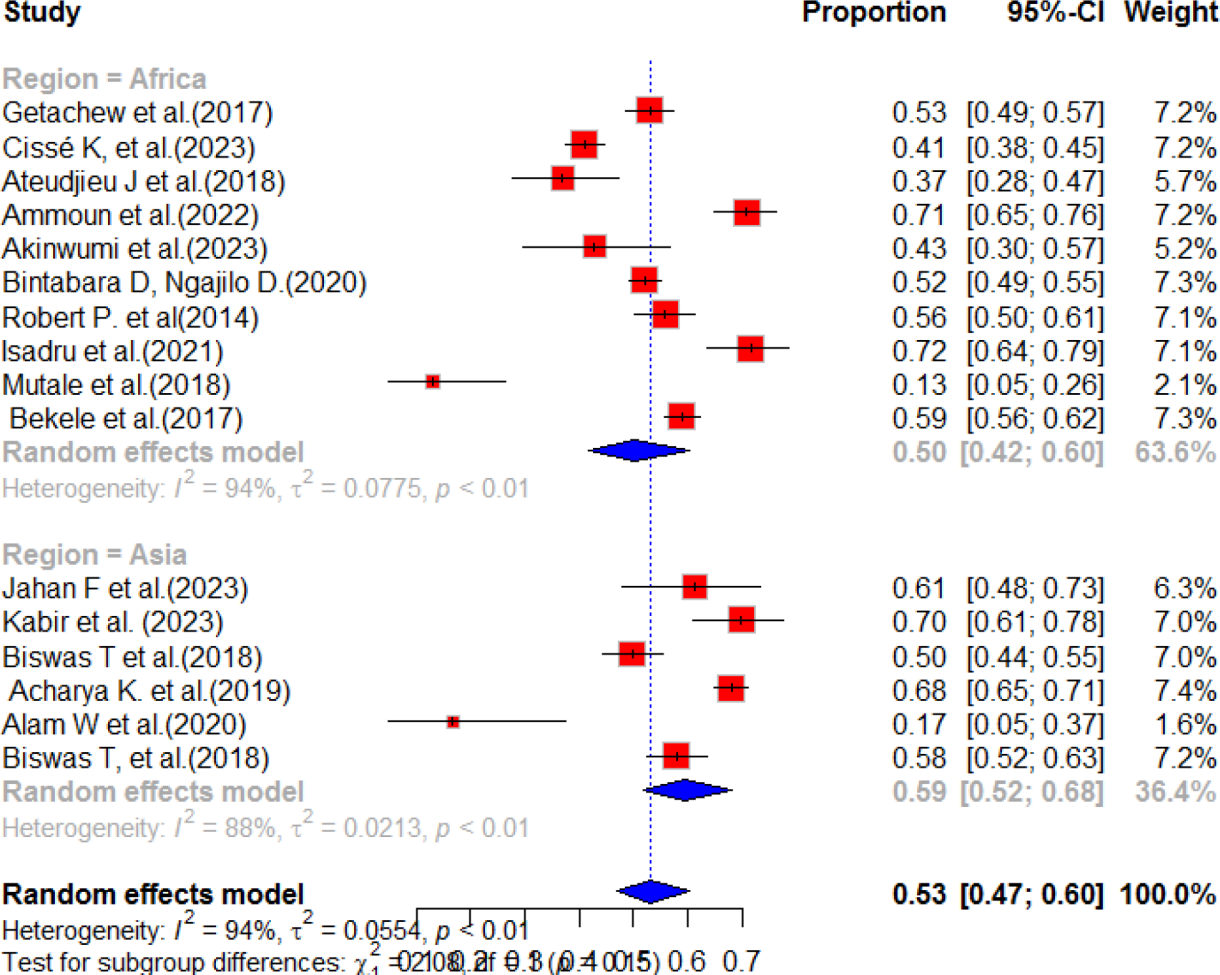
Sub-group analysis of the service preparedness of health facilities to deliver diabetes mellitus services in resource-limited countries.

**Figure 5:**
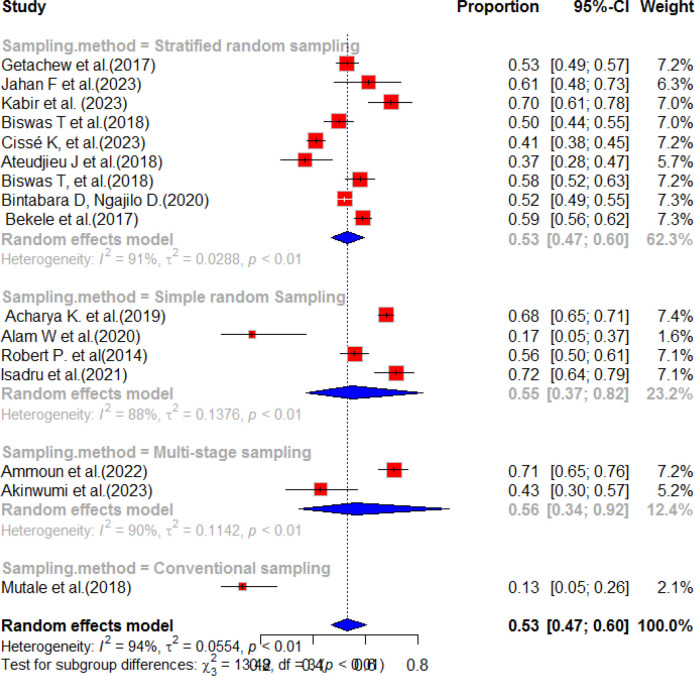
Subgroup analysis by sampling method for service preparedness for diabetes mellitus.

This study examined 10 research studies conducted in Africa and analyzed nine records from Asia to evaluate the preparedness of healthcare services for diabetes mellitus. The results revealed that the pooled prevalence of diabetes mellitus service preparedness in African countries was estimated to be 50.0% (95% CI: 42.0–60.0, I^2^, 94.0%) with a p-value of less than 0.01. This means that, on average, approximately 50% of the required healthcare services for NCDs were prepared in African countries. Similarly, in Asia, the pooled prevalence of diabetes service availability was found to be 59.0% (95% CI: 52.0–68.0, I^2^, 88.0%) with a p-value of less than 0.01 (**Figure 4**). This indicates that, on average, approximately 59.0% of the necessary healthcare services for NCDs were prepared in Asian countries. These findings emphasize the disparities in healthcare service preparedness for NCDs across different regions. While Asia exhibited a higher prevalence of service preparedness compared to Africa, there is still room for improvement in both regions to ensure that the essential healthcare services that individuals with NCDs need are prepared. The subgroup analysis was conducted based on sampling method of each studies. Accordingly, the highest pooled prevalence of diabetes service preparedness was observed in simple random sampling with a pooled prevalence of 55% (95% CI:37.0-82.0) and heterogeneity of 88%, p value<0.01. The second highest pooled prevalence was observed in stratified random sampling method after incorporating nine articles with a pooled result of 53% (95%CI:47-60), I^2^ = 91%, p value ≤ 0.01 (**Figure 5**).

### Pooled prevalence of service availability for diabetes mellitus

**Figure 6:**
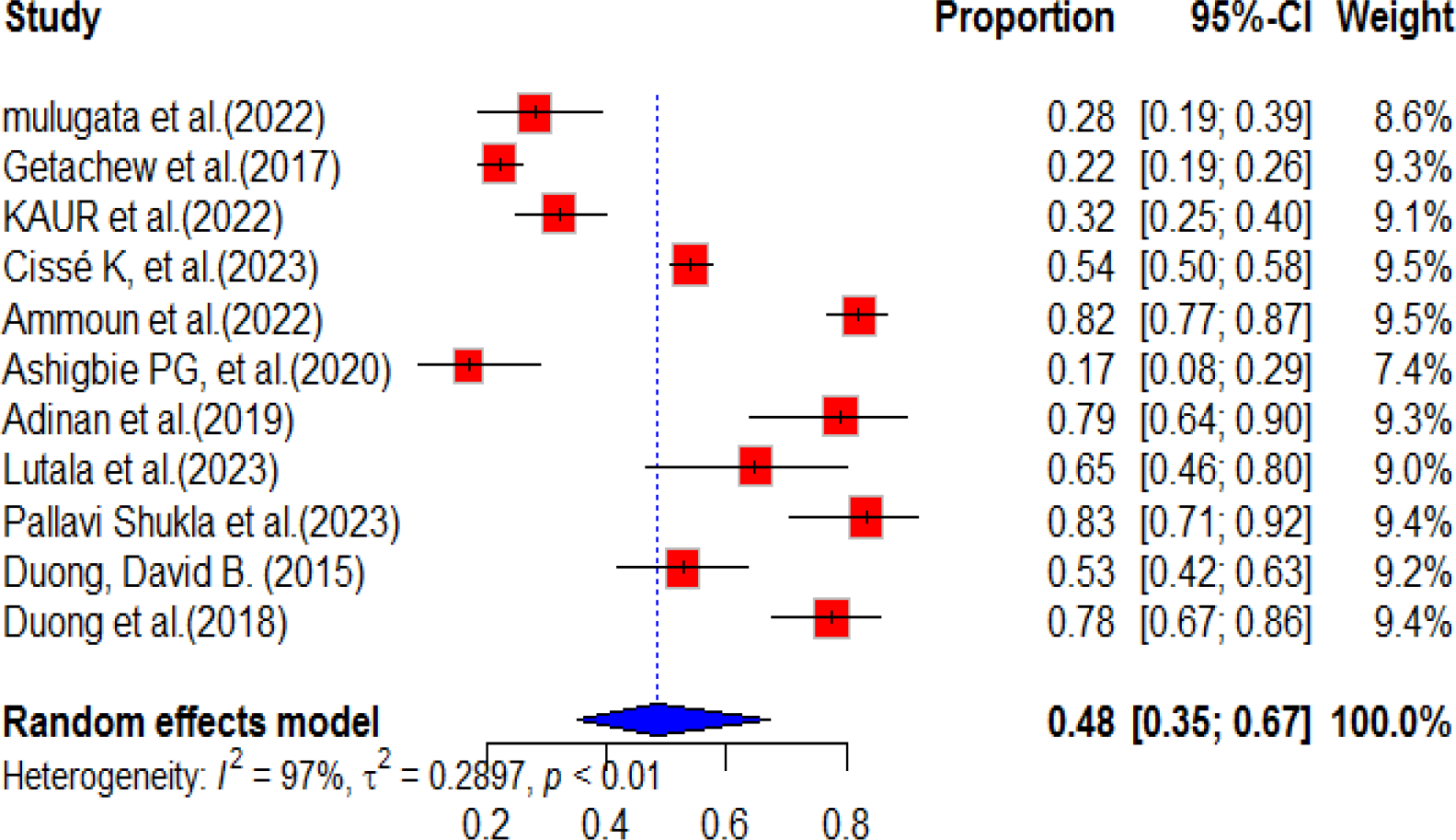
Forest plot of the availability of DM service in health facilities in resource-limited countries.

According to previous literature, the availability of diabetic mellitus health services varies in resource-limited countries. A systematic synthesis of these studies revealed that the overall pooled prevalence of service availability was 48% (95%CI: 36.0, 67.0), with a high level of heterogeneity (97%) and a significant p-value of less than 0.01. The results of this systematic synthesis are presented in **Figure 6**.

### Sub-group analysis of diabetes service availability

**Figure 7:**
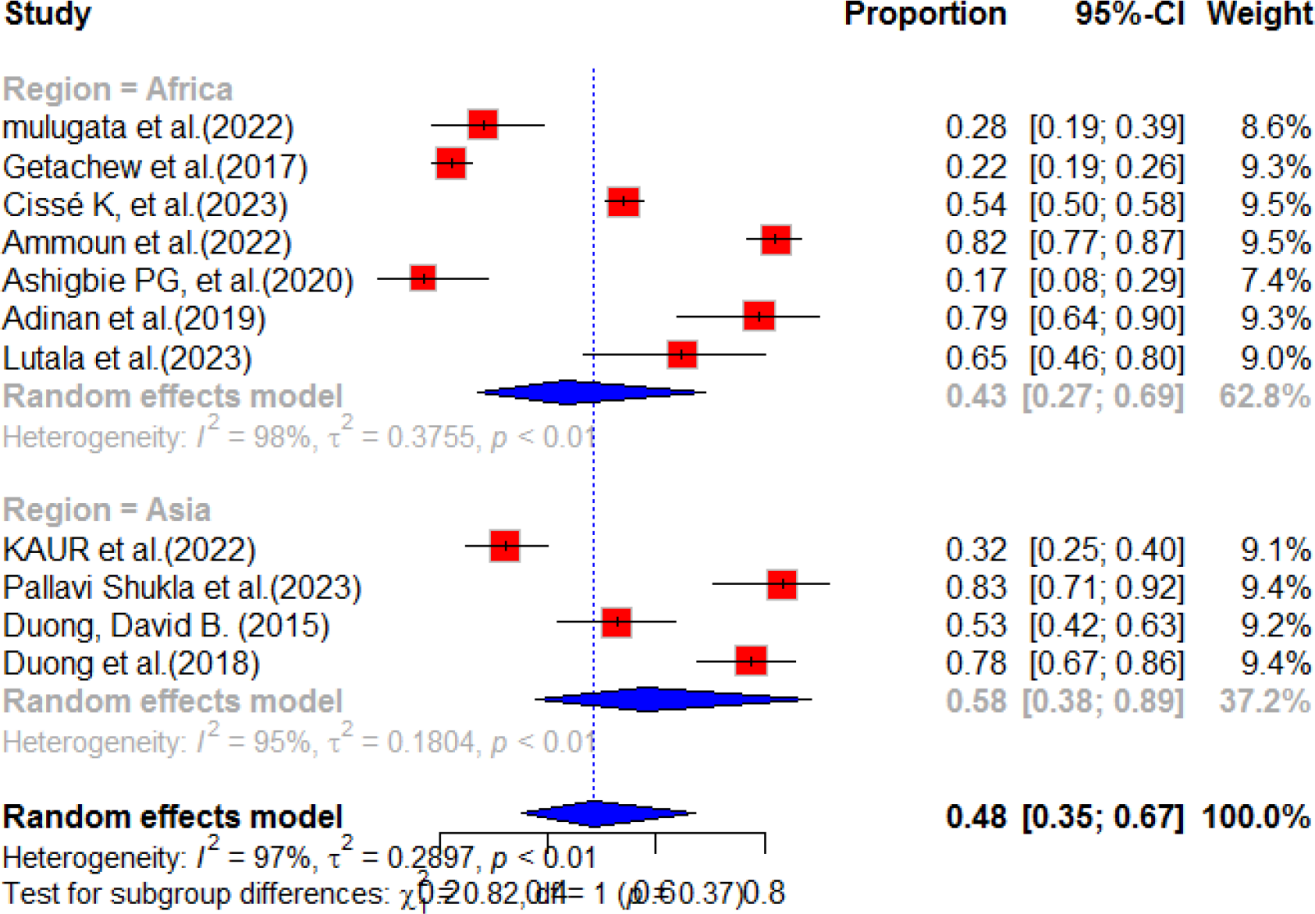
Sub-group analysis forest plot of the availability of diabetes services in health facilities in resource-limited countries.

**Figure 8:**
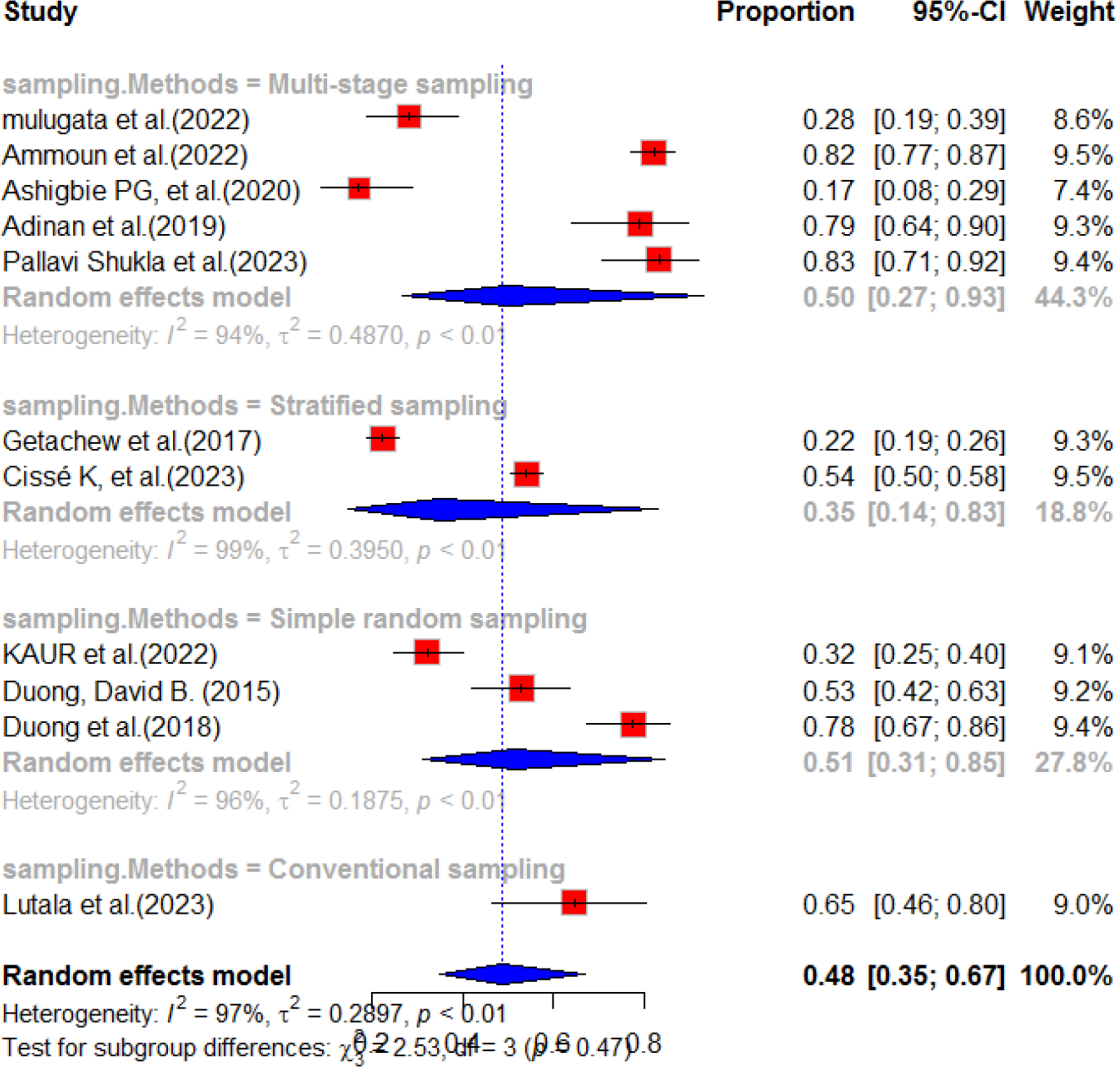
Subgroup analysis by the sampling method of diabetes service availability in resource limited-countries, 2024.

According to a systematic review and meta-analysis, sub-group analyses were conducted for each region included in the individual studies. The highest prevalence of diabetes service availability was observed in Asia, with a pooled prevalence of 58% (95% CI: 38.0-89.0). However, there was a high level of heterogeneity (I^2^ = 95%, p-value<0.01) in this region. In contrast, the pooled prevalence of diabetes mellitus service availability in Africa was relatively low, at 43% (95% CI: 27.0-69.0), with a high level of heterogeneity (98%, p-value<0.01). The overall pooled prevalence of diabetes mellitus service availability in resource-limited countries was found to be 48% (95% CI: 35.0-67.0) (**Figure 7**). A subgroup analysis was conducted based on the sampling method and, based on the finding, five studies were included for multi-stage sampling with a pooled prevalence of 50.0% (95%CI: 27.0-93.0) and heterogeneity of 93%, p value<0.01 (**Figure 8**).

### Meta-bias in prevalence of diabetes service availability

**Figure 9:**
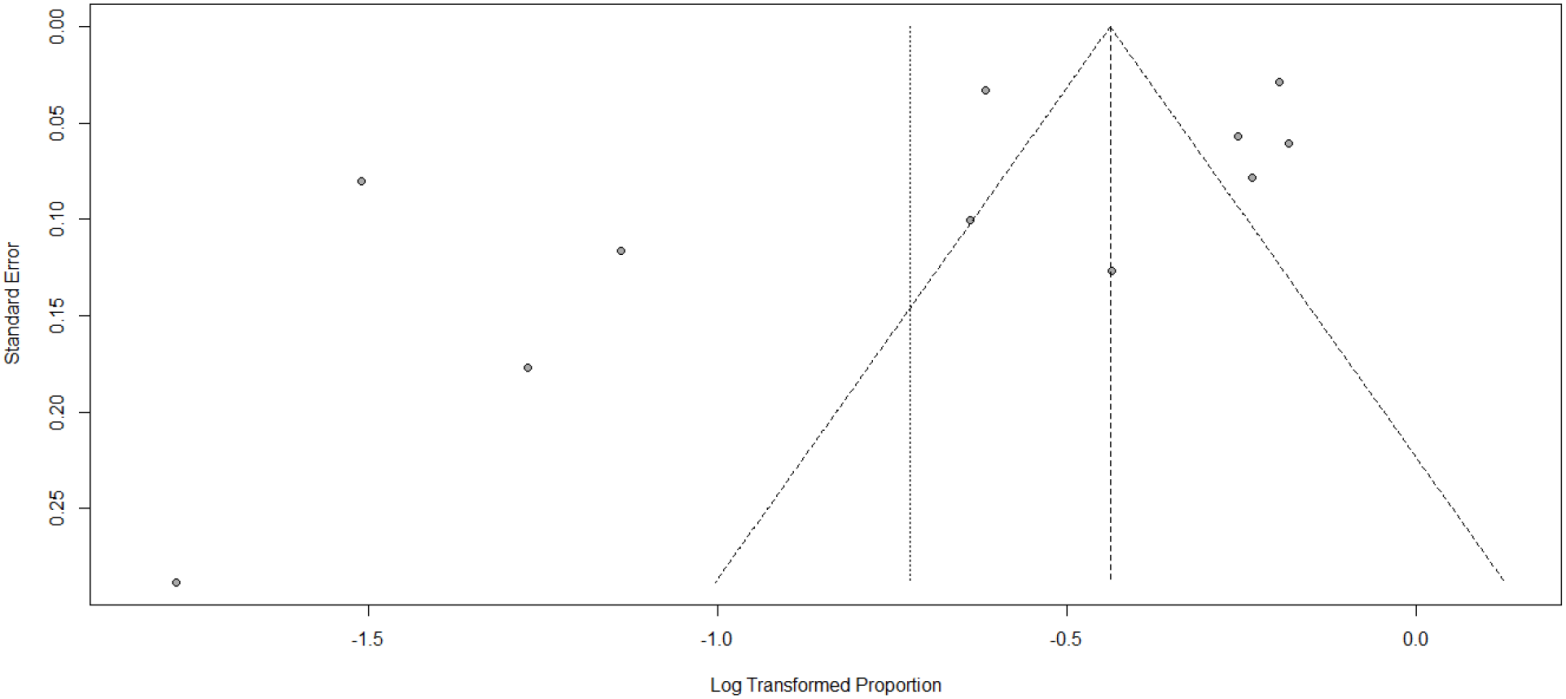
Funnel plot showing publication bias among studies used to compute diabetes service availability in resource-limited countries, 2024.

The publication bias of the studies was checked by using the Egger’s test and funnel plot asymmetry. The Egger’s test of the studies indicated that there is no publication bias. The quantitative heterogeneity of the studies was tau^2^ = 0.6444[0.3386; 1.7139]; tau = 0.8027 [0.5819; 1.309] and I^2^ = 98.1% [97.6%; 98.5%]; H=7.19[6.40; 8.09]. The graphic presentation of this publication bias is presented in funnel plot. The funnel plot of this systematic review indicate there is no visible publication bias (**Figure 9**). The Egger’s test was statistically non-significant with a p-value of 0.1221, with a bias estimate of -5.4489 (SE = 3.1925). Since the Egger’s test was not significant, trim and fill analysis was not necessary to conduct.

### Sensitivity analysis

Sensitivity analysis of the studies was conducted and the result is presented in **Figure 9**. The random effects model was used to calculate a combined estimate, which showed significant variation in the evaluation of the availability of health facilities’ DM services. To address this variation, sensitivity analysis and subgroup analysis were performed. The sensitivity analysis of the study showed all had equal contribution except two studies. These analyses aimed to understand the potential impact of individual studies on the overall estimate of the prevalence of DM service availability. Importantly, the results from the random effects model did not identify any studies that had an excessive influence on the overall estimate. In addition, a sensitivity analysis was also conducted based on subgroup analysis by the sampling method. Nearly all the studies contributed to the pooled result for diabetes service availability. To further investigate the source of heterogeneity, a meta-regression analysis was conducted, considering factors such as sample size and year of publication. However, the results showed that neither sample size nor year of publication had a significant effect on the heterogeneity observed between studies (**Table 4**).

**Table 4:** Meta-regression analysis of factors affecting between-study heterogeneity in the prevalence of availability of diabetes mellitus services in health facilities.

Heterogeneity source	Estimate	Z-value	Std. Err.	p-value	CI	I^2^
Sample size	-0.0004	-0.552	0.0007	0.5809	(-0.0018,0.001)	98.45%
Publication year	0.0337	0.5174	0.0651	0.6048	(-0.0939,0.1613)	98.72%

### Risk of bias evaluation

The Newcastle-Ottawa Scale (NOS) was used to evaluate the quality and potential bias of the studies. This assessment system considers three key parameters: selection, comparability, and outcome. A maximum of 9 points can be assigned, with one author conducting the assessment and another author independently reviewing it to ensure accuracy. The total score determines the level of bias risk, which is categorized as high (less than 5 points), moderate (between 6 and 7 points), or low (between 8 and 9 points) (57) (**Supplementary Table 1**).

## Discussion

This study aimed to assess diabetes service preparedness and availability in resource-limited countries. The meta-analysis revealed that the pooled estimated diabetes service preparedness in healthcare facilities was 53.0%, which is higher than previous studies conducted in Nigeria (58), Burkina Faso (59), and Bangladesh (28). The disparity may be attributed to policies and program variation across the countries regarding diabetes service preparedness in healthcare systems. Other possible explanations for this finding could be variation in health system readiness and some health system only consider the provider perspective. Moreover, the pooled evidence from our study provides stronger support compared to the individual studies conducted in a single area. This highlights the importance of conducting multi-country and multidimensional studies when designing programs and shaping policies for non-communicable disease control. It is evident that in resource-limited countries there are numerous undiagnosed cases of diabetes mellitus, which may be attributed to inadequately prepared health facilities.

The findings of this systematic review on diabetes service preparedness are lower than those of a study conducted in Uganda (32). This difference suggests that health systems can vary significantly between countries. The World Health Organization has also highlighted the variation in health system preparedness across different nations (60). Insufficient preparedness of healthcare facilities to deliver diabetes management services has been identified as a concern (29–35, 38). The possible explanation for this could be that the approaches and the methodologies of health systems may vary from country to country. These findings highlight the need to enhance the readiness of health facilities in low-resource countries to effectively address the escalating diabetes epidemic. The measurement of health system preparedness is universally accepted and recognized worldwide. This indicates that a country’s preparedness is influenced by factors such as political commitment, economic growth, and structural elements. Consequently, addressing the preparedness of diabetes mellitus services requires a multidimensional approach and involvement from multiple sectors.

According to our systematic review and meta-analysis result, the level of health facility preparedness for diabetes mellitus in Africa was found to be 50% (95% CI; 42.0, 60.0). However, in Asia, the pooled prevalence of diabetic service preparedness was higher, at 59% (95% CI: 52.0, 68.0). In comparison, Africa had a lower prevalence of service preparedness for diabetes mellitus. This could be due to Africa being focused on the management of acute infections (61). The study also suggested that the preparedness of diabetic services in different countries and regions is closely linked to their economic growth and health system priority. Therefore, it is crucial for resource-limited countries to focus on service mitigation and integration to address the preparedness problem and reduce the burden of undiagnosed diabetes mellitus in their communities.

Service availability in healthcare is crucial for identifying and managing chronic illnesses in a community. Unfortunately, in 2013, an estimated 174.8 million people worldwide (62) were undiagnosed with diabetes due to various reasons. This systematic review and meta-analysis revealed that the availability of diabetes mellitus services in resource-limited countries was only 48%. This is lower than the World Health Organization’s recommendation that at least 80% (36) of health facilities should have essential medication available. The finding is lower than studies conducted in Kenya (48), Tanzania (30), India (54), and Vietnam (56). The slow pace at which these countries are addressing the growing burden of diabetes is concerning. Inadequate availability of essential anti-diabetic medication has also been reported in other low-resource settings in different countries (29–35, 38). Additionally, while diabetes management guidelines were more likely to be present in health facilities, trained workers were less common. This is alarming because even if protocols are available, they may not be followed if employees are not properly trained. This implies that mitigation health sector transformation with multi-sectorial collaboration is essential to improve the availability of diabetes mellitus services in resource limited countries. In addition, appropriate and mitigated implementation of universal health coverage with strict follow up could improve the availability of the services. Furthermore, working in a balanced way, with both supply-side and demand-side perspectives and the effective implementation of the WHO health system framework, could be improve the availability of diabetes mellitus services in resource-limited setting.

The sub-group analysis indicated that Asia had a higher service availability compared with Africa. While the implementation of universal health coverage (63) has been incorporated all over the world, resource variation and limitation may contribute to the variation of diabetes mellitus service availability. This implies that effective implementation and monitoring of universal health coverage could improve the availability of diabetes services in resource-limited countries.

## Conclusion

The findings of this systematic review and meta-analysis fall below the targets set by the World Health Organization (36). The overall pooled prevalence of service availability and service preparedness were lower than the sustainable development goal of health for all and the WHO’s availability of services (24, 36). The findings highlight the urgent need for interventions and improvements in healthcare services in resource-limited countries. Governments should prioritize the implementation of universal healthcare and primary healthcare to enhance service availability. Investing in health facilities and integrating services for non-communicable and infectious diseases can improve healthcare capacity and preparedness. A tailored and action-oriented approach to health system strengthening is crucial for improving the preparedness and availability of diabetes mellitus services.

## Data availability statement

The raw data supporting the conclusions of this article will be made available by the authors, without undue reservation.

## Author contributions

MA: Conceptualization, Data curation, Formal analysis, Funding acquisition, Investigation, Methodology, Project administration, Resources, Software, Supervision, Validation, Visualization, Writing – original draft, Writing – review & editing. DG: Conceptualization, Formal analysis, Methodology, Project administration, Supervision, Visualization, Writing – original draft, Writing – review & editing. AA: Funding acquisition, Investigation, Methodology, Software, Visualization, Writing – original draft, Writing – review & editing. YN: Data curation, Formal analysis, Methodology, Software, Visualization, Writing – original draft, Writing – review & editing.
