# Supplementary material for: Diabetes mellitus service preparedness and availability: a systematic review and meta-analysis
Source: Front Endocrinol (Lausanne). 2024 Jul 19;15:1427175. doi: 10.3389/fendo.2024.1427175 (PMC11294177; doi:10.3389/fendo.2024.1427175)
Supplement: Supplementary file 1 [file Table_1.docx]

| Authors(Year) | Country | Region | publication year | Sample size | study design | response rate |  | NOS |
| --- | --- | --- | --- | --- | --- | --- | --- | --- |
| Mulugata et al.(2022) | Ethiopia | Africa | 2022 | 82 | cross sectional | 98.80% |  | 7 |
| Getachew et al.(2017) | Ethiopia | Africa | 2017 | 547 | cross sectional | 100.00% |  | 8 |
| KAUR et al.(2022) | India | Asia | 2022 | 156 | cross sectional | 100.00% |  | 7 |
| Cissé K, et al.(2023) | Burkina Faso | Africa | 2023 | 794 | cross sectional | 98.90% |  | 7 |
| Ammoun et al.(2022) | Kenya | Africa | 2022 | 258 | cross sectional | 100.00% |  | 7 |
| Ashigbie PG, et al.(2020) | Kenya | Africa | 2020 | 59 | cross sectional | 100.00% |  | 7 |
| Adinan et al.(2019) | Tanzania | Africa | 2019 | 43 | cross sectional | 100.00% |  | 7 |
| Lutala et al.(2023) | Malawi | Africa | 2023 | 34 | cross sectional | 100.00% |  | 7 |
| Pallavi Shukla et al.(2023) | India | Asia | 2023 | 54 | cross sectional | 100.00% |  | 7 |
| Duong, David B. (2015) | Vietnam | Asia | 2015 | 89 | cross sectional | 100.00% |  | 7 |
| Duong et al.(2018) | Vietnam | Asia | 2018 | 89 | cross sectional | 100.00% |  | 7 |
| Getachew et al.(2017) | Ethiopia | Africa | 2017 | 547 | cross sectional | 100.00% |  | 7 |
| Jahan F et al.(2023) | Bangladesh | Asia | 2023 | 62 | cross sectional | 100.00% |  | 8 |
| Kabir et al. (2023) | Bangladesh | Asia | 2023 | 126 | cross sectional | 100.00% |  | 6 |
| Biswas T et al.(2018) | Bangladesh | Asia | 2018 | 319 | cross sectional | 100.000% |  | 7 |
| Cissé K, et al.(2023) | Burkina Faso | Africa | 2023 | 794 | cross sectional | 98.90% |  | 7 |
| Ateudjieu J et al.(2018) | Cameroon | Africa | 2018 | 100 | cross sectional | 100.00% |  | 7 |
| Acharya K. et al.(2019) | Nepal | Asia | 2019 | 963 | cross sectional | 100.00% |  | 7 |
| Ammoun et al.(2022) | Kenya | Africa | 2022 | 258 | cross sectional | 100.00% |  | 7 |
| Alam W et al.(2020) | Bangladesh | Asia | 2020 | 24 | cross sectional | 100.00% |  | 7 |
| Biswas T, et al.(2018) | Bangladesh | Asia | 2018 | 319 | cross sectional | 100.00% |  | 8 |
| Akinwumi et al.(2023) | Nigeria | Africa | 2023 | 56 | cross sectional | 100.00% |  | 7 |
| Bintabara et al.(2020) | Tanzania | Africa | 2020 | 1188 | cross sectional | 100.00% |  | 7 |
| Robert P. et al(2014) | Tanzania | Africa | 2014 | 335 | cross sectional | 100.00% |  | 7 |
| Isadru et al.(2021) | Uganda | Africa | 2021 | 148 | cross sectional | 100.00% |  | 8 |
| Mutale et al.(2018) | Zambia | Africa | 2018 | 46 | cross sectional | 100.00% |  | 8 |
| Bekele et al.(2017) | Ethiopia | Africa | 2017 | 873 | cross sectional | 100.00% |  | 7 |

Supplementary table 1: Newcastle-Ottawa quality assessment evaluation for cross-sectional study result for this systematic review and meta-analysis
